# The natural history of Perthes' disease: Risk factors in 212 patients followed for 5 years

**DOI:** 10.3109/17453674.2010.533935

**Published:** 2010-11-26

**Authors:** Terje Terjesen, Ola Wiig, Svein Svenningsen

**Affiliations:** ^1^Department of Orthopedics, Oslo University Hospital, Rikshospitalet, Oslo; ^2^Department of Orthopedics, Sørlandet Hospital, Arendal, Norway

## Abstract

**Background:**

The prognosis in Perthes' disease varies considerably according to certain risk factors, but there is no concensus regarding the relative importance of these factors. We assessed the natural history of the disease and defined prognostic factors of value in deciding the proper treatment.

**Patients and methods:**

During the 5-year period 1996–2000, a nationwide study on Perthes' disease was performed in Norway. 425 patients were registered. The present study involved the 212 children (mean age 5.1 years, 77% boys) who were affected unilaterally and who had been treated with physiotherapy only (which is considered not to change the natural history). They were followed by taking radiographs at the time of diagnosis and after 1, 3, and 5 years. At the 5-year follow-up, the outcome was evaluated according to a modification of the Stulberg classification: good (spherical femoral head), fair (ovoid femoral head), and poor (flat femoral head).

**Results:**

The 5-year radiographic results were strongly dependent on 4 risk factors: age 6 years or more at diagnosis, total femoral head necrosis, height of the lateral pillar of the epiphysis less than 50% of normal height, and femoral head cover less than 80%. As the number of risk factors increased from 0 to 4, the proportion of patients with good radiographic 5-year outcome decreased from 79% to 0% and the proportion with poor outcome increased from 3% to 91%.

**Interpretation:**

Most children under 6 years of age do not need any special treatment. In older children, no special treatment is indicated if the whole femoral head is not necrotic and the femoral head cover is > 80%. In the most severe forms of the disease (i.e. more than 2 risk factors), surgical containment treatment seems advisable.

The prognosis in Perthes' disease varies considerably according to risk factors such as age, the amount of femoral head involvement, and femoral head cover (Catterall 1971, Green et al. 1981, Stulberg et al. 1981, Herring et al. 1992). Studies on prognosis have to account for these risk factors and the number of patients has to be large to make the statistical analysis of subgroups meaningful and reliable. These requirements were met by the Norwegian nationwide prospective study on Perthes' disease in which 368 children with unilateral affection were included (Wiig et al. 2008). A large proportion of the patients were managed by physiotherapy only. Since this treatment has never been proven to have any effect on outcome, we believe that it reflects the natural history of the disease. This provided a good opportunity to study risk factors in detail. Such information would be useful in clinical practice to distinguish between children who only need symptomatic treatment and those with a poor prognosis, for whom surgical treatment should be considered.

We wanted to determine the radiographic development and natural history of Perthes' disease, and to define risk factors that could be used in deciding optimal treatment.

## Patients and methods

During the 5-year period 1996–2000, a nationwide study on Perthes' disease was performed in Norway. All hospitals with pediatric orthopedic service (6 university hospitals, 16 county hospitals, and 6 local hospitals) reported every new incident of Perthes' disease. Recruitment to the study was done by obtaining informed consent, and it was approved by the Norwegian Data Inspectorate and the Norwegian Directorate of Health and Social Affairs.

Children with less than 50% femoral head necrosis (Catterall group 1 or 2) received physiotherapy as the only treatment. Patients with more than 50% necrosis (Catterall group 3 or 4) and aged 6 years or older at diagnosis, received either physiotherapy, Scottish Rite abduction orthosis, or proximal femoral varus osteotomy, according to the choice of the local orthopedic surgeons. The decision was based on the surgeons' preferences and treatment philosophy, and all patients from the same hospital were treated by the same method. Children under 6 years of age with more than 50% necrosis and adequate femoral head cover (> 80%) were treated with physiotherapy. Those with markedly reduced head cover in the fragmentation phase were allocated to the same treatment groups as described for older children.

425 patients were registered. 217 children were affected unilaterally and had been treated with physiotherapy only. Radiographic 5-year follow-up was lacking in 5 patients. Thus, the present study comprised 212 children.

Physiotherapy consisted of range-of-movement exercises and muscle strengthening exercises, in addition to advice and support from the physiotherapist to the children and parents. Depending on the range of motion, the training sessions took place from once a week to once a month. In addition, the children were instructed to perform exercises at home.

At the 5-year follow-up, the patients and their parents were asked about pain, limping gait, and level of function. Walking distance was considered normal if the patient could walk 5 km; otherwise, it was classified as reduced. Sporting activity was classified into 4 categories: normal, participation in all activities but with reduced function, activities such as swimming only, and no participation.

Radiographs included an anteroposterior view with the patient in the supine position, with legs parallel and in neutral rotation, and a Lauenstein projection with the legs flexed, abducted, and externally rotated. Radiographs were taken at the time of diagnosis and at the follow-up examinations 1 year, 3 years, and 5 years after diagnosis. The radiographs were evaluated and measured by a pediatric orthopedic surgeon (SS) with great experience in examining radiographs of hips in children.

The amount of femoral head necrosis was classified according to Catterall (1971). Group 1 means affection of only a small part of the anterior epiphysis and group 2 means affection of up to 50% of the femoral head. In group 3, more than 50% is affected and in group 4 the whole epiphysis is involved. Because the correct amount of involvement can be unreliable at the initial stage of the disease, the Catterall grouping was based on both the initial radiographs and those at the 1-year follow-up. If involvement was more severe after 1 year, the highest Catterall group was used.

We also used the lateral pillar classification by Herring et al. (1992): group A with no reduction in the height of the lateral pillar of the femoral head, group B with more than 50% of the height of the lateral pillar maintained, and group C with reduction of the lateral pillar by more than 50%. The initial radiographs and those after 1 year were used in the same way as for the Catterall grouping.

The femoral head cover was determined by calculating the percentage of the femoral head medial to Perkins' line in relation to the width of the femoral head parallel to Hilgenreiner's line, as described by Heyman and Herndon (1950). The articulotrochanteric distance (ATD) was measured as the distance between 2 lines parallel to Hilgenreiner's line—the line through the tip of the greater trochanter and that through the most proximal point of the femoral head.

At the 5-year follow-up, the hips were classified according to Stulberg et al. (1981) as modified by Neyt et al. (1999). The Stulberg classification, which originally contained 5 classes, was modified into a 3-group classification, where hips in group A (Stulberg classes I and II) had spherical femoral heads, those in group B (Stulberg III) had ovoid femoral heads, and hips in group C (Stulberg classes IV and V) had flat outlines of the femoral head. Group A hips have a good radiographic outcome because no osteoarthritis was seen in such hips after a follow-up time of 30–40 years (Stulberg et al. 1981). Because approximately half of the hips in group C had developed osteoarthritis with narrowing of the joint space, these hips represent a poor radiographic result. Group B represents a fair radiographic outcome.

### Statistics

Categorical variables were analyzed by cross-tables with the chi-square test. Continuous variables were analyzed by the t-test for independent samples. Pearson's correlation coefficient (r) was used for correlation between continuous variables. Differences were considered significant when the p-value was < 0.05.

## Results

Of the 212 children included, there were 164 boys (77%) and 48 girls. Mean age was 5.1 (1.3–12) years. 59 children were older than 6.0 years and 22 children (10%) were older than 8.0 years. The left hip was affected in 118 patients (56%) and the right hip in 94. The mean duration of symptoms (pain and limping gait) prior to diagnosis was 4.6 (0–24) months, and was similar in girls and boys. Children older than 6.0 years had a longer duration of symptoms than younger children (6.5 vs. 3.9 months; p = 0.004).

The radiographs at the time of diagnosis showed that 55% of the hips were at the initial stage, 44% were at the fragmentation stage, and 1% were at the reossification stage. At the 1-year follow-up, 56% of the hips were at the fragmentation stage and 43% were at the reossification stage. The degree of femoral head affection was Catterall group 1 in 9 patients, group 2 in 28 patients, group 3 in 84, and group 4 in 91 patients. Thus, 83% of the patients had necrosis of more than half of the femoral head. There was no statistically significant association between Catterall distribution and sex; nor was there a greater proportion of total femoral head involvement in patients older than 6.0 years than in younger children.

The distribution of hips according to lateral pillar height was group A in 8 patients, group B in 124 patients, and group C in 80 patients. There were no statistically significant differences according to age and sex. There was a strong association (p < 0.001) between the Catterall classification and the lateral pillar classification (Table 1). The most frequent combinations were Catterall 3/lateral pillar B (35% of the hips) and Catterall 4/lateral pillar C (33%).

**Table 1.** Relationship between the Catterall classification and the lateral pillar classification (number of patients)

Catterall classification	Lateral pillar classification			Total
	group A	group B	group C	
Group 1	5	4	0	9
Group 2	3	25	0	28
Group 3	0	74	10	84
Group 4	0	21	70	91
Total	8	124	80	212

The mean femoral head cover (FHC) of the normal hips was 98% at the time of diagnosis in children under 6 years and 96% in older children, and it decreased 4–5% during the 5-year period (Table 2). The lower limit of the normal range (mean – 2 SD) was about 80%.

**Table 2.** Femoral head cover (%) of the normal hip at the time of diagnosis, and after 1, 3, and 5 years

Time	Femoral head cover					
	Age < 6.0 years			Age > 6.0 years		
	Mean	SD	LL	Mean	SD	LL
At diagnosis	98	7.4	83	96	6.1	84
1-year follow-up	98	6.2	86	95	5.5	84
3-year follow-up	96	5.9	84	94	7.8	78
5-year follow-up	93	6.6	80	92	7.4	77

LL: Lower limit of normal range (mean – 2 SD)

During the 5-year period, FHC of the Perthes' hips decreased by 4–5% in hips with femoral head necrosis of less than 50% (Table 3). A considerably greater reduction, 11–15%, occurred in hips with more than 50% affection. The reduction was most pronounced during the first year after diagnosis, but continued to the 3-year follow-up (p < 0.001). Thereafter, no significant change occurred. FHC at the time of diagnosis and that at the 5-year follow-up correlated (r = 0.26, p < 0.001) as did FHC at the 1-year follow-up and at the 5-year follow-up (r = 0.26, p < 0.001). FHC was similar in girls and boys.

**Table 3.** Femoral head cover (%) of the hip with Perthes' disease at the time of diagnosis, and after 1, 3, and 5 years, according to age (years) and degree of femoral head necrosis

	Femoral head cover							
	Catterall groups 1 and 2				Catterall groups 3 and 4			
	Age < 6.0		Age > 6.0		Age < 6.0		Age > 6.0	
Time	Mean	SD	Mean	SD	Mean	SD	Mean	SD
At diagnosis	93	7.6	93	9.8	94	12	87	12
1-year follow-up	92	8.7	92	8.7	83	11	78	7.2
3-year follow-up	89	9.8	**^a^**		79	9.7	75	9.5
5-year follow-up	89	8.7	88	5.8	79	11	76	9.6

**^a^** only 2 patients.

The mean articulotrochanteric distance (ATD) of the normal hips was 22 mm at the time of diagnosis and 20 mm at the 5-year follow-up. The mean ATD of the affected hips decreased from 21 mm at the time of diagnosis to 18 mm at the 5-year follow-up in Catterall group 1 and 2 hips (p = 0.007), and from 20 mm to 15 mm in Catterall 3 and 4 hips (p < 0.001).

At the 5-year follow-up, 155 patients had no pain, whereas pain in the hip, thigh, or knee was reported by 54 patients (25%), 46 of whom had slight pain only and 8 of whom had more pronounced complaints. Limping gait was reported in 49 patients (23%). Reduced walking distance was reported by 29 children (14%). 46 children (22%) had reduced physical function and 5 of them could only take part in activities like swimming. The clinical examination showed that 60 children had a mean leg length inequality of 11 mm (SD 6.6, range 5–40) with the affected leg shortest. There were strong associations between the radiographic 5-year outcome and the clinical parameters pain, limp, walking capacity, and physical activities, with more complaints and worse function in hips with poor radiographic outcome.

**Figure 1.**
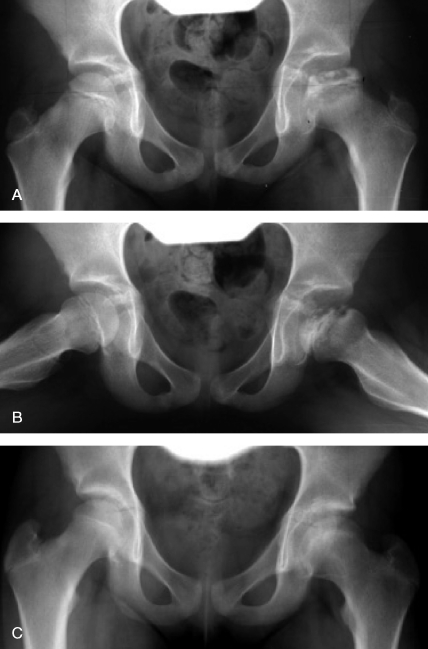
A 7-year-old girl with Perthes' disease of the left hip. She had symptoms for 1 month before diagnosis (pain and limping gait). A and B. At 1-year follow-up, showing Perthes' disease classified as Catterall group 3 and lateral pillar group B. C. At 5-year follow-up, showing good radiographic outcome with spherical femoral head.

The radiographic outcome at the 5-year follow-up was good (i.e. spherical femoral head) in 114 patients (54%), fair (ovoid femoral head) in 60 children, and poor (flat femoral head) in 38 children (18%). There was a strong relationship between radiographic outcome and Catterall group, as no hips with < 50% femoral head necrosis had a poor outcome—whereas 37% of those with total necrosis had a poor result (Table 4). The outcome was good in two-thirds of the hips in Catterall group 3 (Figure 1) and only 4% had a poor result. The same trend of worse radiographic outcome with greater femoral head involvement occurred when using the lateral pillar classification. All the hips in group A and two-thirds in group B had good radiographic outcome, whereas this was seen in only one quarter of the hips in lateral pillar group C. We found no significant association between gender and radiographic outcome (p = 0.1).

**Table 4.** Radiographic 5-year outcome (modified Stulberg classification) in relation to the Catterall classification and lateral pillar classification

	Radiographic outcome					
	Good		Fair		Poor	
Classification	n	%	n	%	n	%
Catterall						
Group 1	9	100	0	0	0	0
Group 2	23	82	5	18	0	0
Group 3	56	67	24	29	4	4
Group 4	26	29	31	34	34	37
Lateral pillar						
Group A	8	100	0	0	0	0
Group B	85	68	28	23	11	9
Group C	21	26	32	40	27	34

n: number of patients; %: percentage of patients.

There was a clear association between the radiographic outcome and the age of the patients at the time of diagnosis. Younger children had better results than older children, no matter whether the age limit was 5 years, 6 years, or 7 years (p = 0.001 to 0.002). The differences between the age groups were significant when all the hips were examined as well as when only Catterall 3 and 4 hips were included. The mean age of patients with a good 5-year radiographic outcome was 4.8 years and it was 6.1 years in those with a poor result (p < 0.001). When analyzing children over 6.0 years in Catterall groups 3 and 4, there were no significant differences in age between patients with good and poor radiographic outcome (7.5 and 7.6 years; p = 0.7); nor was there any significant difference when those under 6 years were analyzed (p = 0.2).

**Figure 2.**
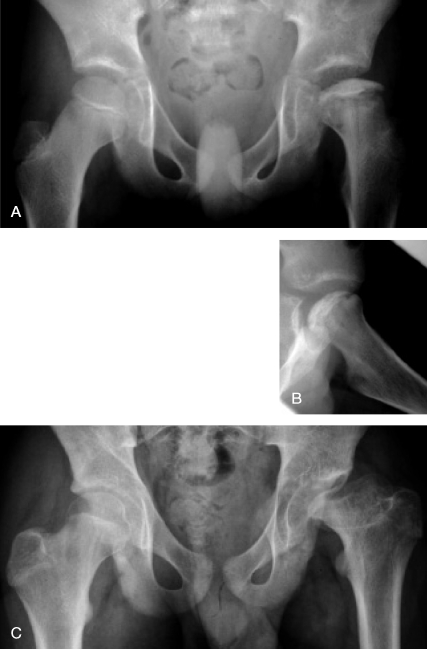
A 9-year-old boy with Perthes' disease of the left hip. He had had pain in his left knee and limping for 2 months prior to diagnosis. A and B. At the time of diagnosis, showing Perthes' disease classified as Catterall group 4 and lateral pillar group C. C. At 5-year follow-up, showing poor radiographic outcome with flattened left femoral head.

In children under 6 years of age, the results in Catterall group 3 were considerably better than those in group 4 (Table 5). In children over 6.0 years of age, the difference was even more pronounced with poor results in 7% of Catterall group 3 hips and 75% in group 4 hips (Figure 2). There was a similar trend of better results in lateral pillar group B than in group C (Table 5).

**Table 5.** Radiographic 5-year outcome (modified Stulberg classification) in relation to age and femoral head affection in patients with more than 50% femoral head necrosis

	Radiographic outcome											
Femoral head affection	Age < 6.0 years						Age > 6.0 years					
	Good		Fair		Poor		Good		Fair		Poor	
	n	%	n	%	n	%	n	%	n	%	n	%
Catterall												
Group 3	40	70	15	26	2	4	16	59	9	33	2	7
Group 4	25	37	26	39	16	24	1	4	5	21	18	75
Lateral pillar												
Group B	45	68	14	21	7	11	16	55	9	31	4	14
Group C	20	34	27	47	11	19	1	5	5	23	16	72

n: number of patients; %: percentage of patients.

The radiographic outcome in children with necrosis of the whole femoral head (Catterall group 4) deteriorated with increasing age (p < 0.001). In children under 6 years of age, there were more good results than poor results whereas in those over 6.0 years as many as 75% of the patients had poor results (Table 6).

**Table 6.** Radiographic 5-year outcome in relation to age at diagnosis in children with total femoral head involvement (Catterall group 4)

Age at diagnosis (year)	Radiographic outcome						
	Good		Fair		Poor		Total
	n	%	n	%	n	%	n
< 4	16	41	18	46	5	13	39
4.0–5.9	9	32	8	29	11	39	28
6.0–7.9	1	6	3	19	12	75	16
> 8.0	0	0	2	25	6	75	8

n: number of patients; %: percentage of patients.

In patients with more than 50% femoral head necrosis, there was no statistically significant association between FHC at the time of diagnosis and radiographic 5-year results—either in children under 6 years of age (p = 0.3) or in older children (p = 0.2). At the 1-year follow-up, the FHC was lower in hips with a poor radiographic outcome than in those with a good outcome in children above 6.0 years of age (85% vs. 75%; p = 0.007) and in younger children (87% vs. 80%; p = 0.007).

In an attempt to make a more thorough evaluation of the prognosis in patients with more than 50% femoral head necrosis, the significant risk factors were combined: Catterall group, lateral pillar group, age at diagnosis, and femoral head coverage. Based on the results of this study, 4 risk factors were defined: Catterall group 4, lateral pillar group C, age at diagnosis of 6 years or more, and FHC of less than 80% at the 1-year follow-up. Each patient was scored according to these criteria and the number of risk factors varied from 0 to 4. Since the dividing line regarding age was 6.0 years, young children could have 0–3 risk factors while older children could have 1–4 risk factors. The association between risk factors and radiographic outcome is shown in Table 7. When 4 risk factors were identified, there were no good radiographic results and there were 91% poor results. With decreasing number of risk factors, the chance of having a good radiographic outcome increased.

**Table 7.** Radiographic 5-year outcome (modified Stulberg classification) according to the number of risk factors and age at diagnosis in patients with more than 50% femoral head necrosis

	Radiographic outcome (modified Stulberg classification)											
	Age < 6.0 years (n = 124)						Age > 6.0 years (n = 51)					
Number of risk factors **^a^**	Good		Fair		Poor		Good		Fair		Poor	
	n	%	n	%	n	%	n	%	n	%	n	%
0	30	79	7	18	1	3						
1	15	65	6	26	2	9	10	67	4	27	1	7
2	15	35	18	43	9	21	7	64	3	27	1	9
3	5	24	10	48	6	28	0	0	6	43	8	57
4							0	0	1	9	10	91

**^a^** Risk factors: Catterall group 4, lateral pillar group C, age over 6.0 years, and femoral head cover of < 80%.

Compared to younger children, children over 6.0 years of age had a worse prognosis (Table 7); there were no good radiographic results when the number of risk factors was more than 2. With 1 or 2 risk factors, good results were seen in about two-thirds of the hips. Poor outcome increased from less than 10% to 91% as the number of risk factors increased from 1 to 4.

## Discussion

The overall 5-year radiographic outcome according to treatment and risk factors in the Norwegian nationwide study on Perthes' disease has been published previously (Wiig et al. 2008). The outcome was better after proximal femoral varus osteotomy than after physiotherapy in the “worst” prognostic group (patient age of more than 6.0 years at diagnosis and femoral head necrosis of more than 50%). This could lead to the simple conclusion that all children in this group should undergo surgical treatment. However, one third of those treated nonoperatively had good final outcome. If these children had been operated, it would have been unnecessary and potentially harmful because of the risk of complications. The aim of the present study was to evaluate those children who received only symptomatic treatment (including physiotherapy) in greater detail, to identify those who had a good radiographic outcome—in order to avoid operation. On the other hand, risk factors for a poor outcome should be defined early in the disease, to give such children an opportunity to have more active treatment aimed at improving containment and outcome.

Classification of the degree of femoral head necrosis into 4 groups was introduced by Catterall (1971), who reported that the chances of a good radiographic outcome decreased from group 1 to group 4. This has been confirmed in several studies (Dickens and Menelaus 1978, Kelly et al. 1980, Green et al. 1981, Mukherjee and Fabry 1990). Although we found the same, it was somewhat surprising that the greatest difference in outcome was between Catterall groups 3 and 4. Actually, the results in group 3 were satisfactory with good radiographic outcome in two-thirds of the hips and poor outcome in only 4%. This shows that Catterall groups 3 and 4, although sometimes difficult to differentiate, should be dealt with individually as risk factors.

The lateral pillar classification is also based on the amount of femoral head affection (Herring et al. 1992). Marked reduction of the pillar to less than half of its normal height (group C), represents a severe form of Perthes' disease. According to Herring et al. (1992), the lateral pillar classification is in part a modification of the Catterall classification. This is in line with our results, which showed a close association between the two classifications. The lateral pillar classification was revised a few years ago (Herring et al. 2004), introducing a new group: “B/C border”. We have no experience with the revised version, but we find it rather difficult to understand that a classification that was introduced because of problems in distinguishing between groups B and C should be more reliable when an additional category is added.

Our findings have confirmed the experience of others (Herring et al. 1992, Ismail and Macnicol 1998) that the lateral pillar classification is of considerable prognostic significance. In patients over 6.0 years of age and with more than 50% femoral head involvement, we found markedly better results in lateral pillar group B hips than in group C hips. Thus, lateral pillar group C should be considered a risk factor.

Age at diagnosis is strongly related to final outcome (Catterall 1971, Kelly et al. 1980, Stulberg et al. 1981, Ippolito et al. 1987, Herring et al. 1992, Ismail and Macnicol 1998). We found the same, with a better final outcome in younger children. It has not been wholly clarified what age is the most adequate dividing line relative to prognosis. Poor results in patients over 9 years of age have been reported (Ippolito et al. 1987, Herring et al. 1992). This was confirmed by us in Catterall group 4 (all 4 patients had poor results) whereas Catterall group 3 had better results. Weinstein (1988) wrote that “8 years seems to be the watershed”. This is hardly in agreement with our study, because the outcome in patients with more than 50% femoral head involvement was not worse in patients over 8.0 years of age than in those aged 6.0–7.9 years. Our results in the worst prognostic group (Catterall 4) indicate that the most useful limit relative to prognosis is 6 years because the greatest difference in outcome was between the the age groups 4.0–5.9 years and 6.0–7.9 years. We nevertheless agree with Fabry et al. (2003) that “young age is not a free ticket to a good result” and several of our children under 6 years of age had a poor radiographic outcome. However, the conclusion remains that younger children—and especially those under 4 years—definitely have a better prognosis.

Reduced femoral head cover is an important factor in the development of deformation of the femoral head (Catterall 1971, Green et al. 1981, Joseph et al. 2003). Our results show a marked reduction in the cover during the first year, and at the 1-year follow-up it was lower in hips with poor 5-year outcome than in hips with good outcome. According to Green et al. (1981), epiphyseal extrusion (the same as reduced femoral head cover) of more than 20% indicates a poor prognosis. In line with this, Gu and Da Paz (1999) maintained that operative containment may be needed for symptomatic patients whose cover is less than 80%. Our results also indicate a dividing line at 80% for two reasons. First, the lower normal limit of normal hips (mean – 2 SD) was approximately 80%. Secondly, the mean cover 1 year after diagnosis in hips with a poor radiographic outcome was lower than 80%. Consequently, we consider a cover of less than 80% to be a risk factor.

As opposed to the other risk factors, reduced femoral head cover can be influenced by treatment. “Containment” methods such as femoral varus osteotomy will increase the cover and reduce the risk of deformation of the epiphysis. Joseph et al. (2003) reported that more than 20% extrusion occurred in 70% of the hips by the stage of early reossification and that if containment were to succeed, it should be achieved before this stage. The duration of the fragmentation stage is approximately 9 months (Joseph et al. 2003). According to our results, the greatest reduction in FHC occurred during the first year and many hips were still at the fragmentation stage at the 1-year follow-up. Radiographs should be taken every 3 or 4 months to follow the radiographic stages and the degree of uncovering, and containment surgery should be considered in children with worsening prognostic signs during the first year.

Although Catterall (1971) stated that the involvement groups did not change with time, others have found that correct staging could not be definitely determined for a period of up to 8 months after the initial radiographs (Dickens and Menelaus 1978, Kelly et al. 1980). Moreover, the Catterall classification is not always easy to apply and some authors have reported a considerable interobserver variation (Christensen et al. 1978, Hardcastle et al. 1980). We therefore think that it would be better to use a multifactorial prognostic approach.

Combinations of risk factors have been proposed previously. A 3-factor combination with age, lateral extrusion, and amount of epiphyseal involvement has been used by some (Green et al. 1981, Mukherjee and Fabry 1990, Kamegaya et al. 2005). MRI was used by Sanctis and Rondinella (2000), who recommended a combination of extent of necrosis and 2 MRI risk signs: lateral extrusion and physeal involvement. We think that plain radiography is sufficient when using 4 risk factors: necrosis of the whole epiphysis, lateral pillar group C, age 6 years or more, and femoral head cover of less than 80%. By adding these signs, each patient obtained a score that proved suitable for prediction of the prognosis. We recommend this method, which is relatively simple and reliable. When 4 risk factors are used, the consequence of making an error in one factor need not violate the total evaluation.

What are the consequences of our study for clinical practice? Patients with less than 50% femoral head necrosis do not need any special treatment. In children under 6 years of age with more than 50% femoral head affection, containment treatment such as femoral varus osteotomy is rarely indicated, even if the whole epiphysis is involved, unless femoral head cover decreases towards subluxation. In children over 6.0 years of age, symptomatic treatment alone is indicated if the whole epiphysis is not necrotic and the femoral head cover remains satisfactory (i.e. over 80%). In the most severe forms of Perthes' disease (with 3–4 risk factors), containment surgery seems advisable. This means that the great majority of the children in the present study had an adequate non-containment treatment, whereas those with the most severe form of the disease and relatively older age would probably have had a better outcome if containment surgery had been performed.
